# Congestion Transition in Air Traffic Networks

**DOI:** 10.1371/journal.pone.0125546

**Published:** 2015-05-20

**Authors:** Bernardo Monechi, Vito D. P. Servedio, Vittorio Loreto

**Affiliations:** 1 Sapienza University of Rome, Physics Dept., Piazzale Aldo Moro 2, 00185 Roma, Italy; 2 Institute for Complex Systems (ISC-CNR), Via dei Taurini 19, 00185 Roma, Italy; 3 Institute for Scientific Interchange (ISI), Via Alassio 11/C, 10126 Torino, Italy; 4 SONY-CSL, 5, Rue Amyot, 75005, Paris, France; Beihang University, CHINA

## Abstract

Air Transportation represents a very interesting example of a complex techno-social system whose importance has considerably grown in time and whose management requires a careful understanding of the subtle interplay between technological infrastructure and human behavior. Despite the competition with other transportation systems, a growth of air traffic is still foreseen in Europe for the next years. The increase of traffic load could bring the current Air Traffic Network above its capacity limits so that safety standards and performances might not be guaranteed anymore. Lacking the possibility of a direct investigation of this scenario, we resort to computer simulations in order to quantify the disruptive potential of an increase in traffic load. To this end we model the Air Transportation system as a complex dynamical network of flights controlled by humans who have to solve potentially dangerous conflicts by redirecting aircraft trajectories. The model is driven and validated through historical data of flight schedules in a European national airspace. While correctly reproducing actual statistics of the Air Transportation system, e.g., the distribution of delays, the model allows for theoretical predictions. Upon an increase of the traffic load injected in the system, the model predicts a transition from a phase in which all conflicts can be successfully resolved, to a phase in which many conflicts cannot be resolved anymore. We highlight how the current flight density of the Air Transportation system is well below the transition, provided that controllers make use of a special re-routing procedure. While the congestion transition displays a universal scaling behavior, its threshold depends on the conflict solving strategy adopted. Finally, the generality of the modeling scheme introduced makes it a flexible general tool to simulate and control Air Transportation systems in realistic and synthetic scenarios.

## Introduction

Air transportation is one of the most important and fast ways of traveling in present days. The possibility of connecting distant areas at relatively affordable cost, makes this transport the best-suited for continental and inter-continental trips. Despite the competition with other growing transportation systems, mainly high-speed railways, and the recent economical crisis that reduced the overall load of traffic, an increase of air traffic demand over Europe and USA is foreseen in the coming years [1, 2]. This increase of traffic load could bring the current Air Traffic Management (ATM) system over its capacity limits, so that safety and performance of every flight could not be guaranteed anymore. Even in the present situation dangerous safety occurrences are not unlikely to happen even though they are very rare events. EUROCONTROL [3], an international organization composed of Member States of the European Region dealing with almost every aspect of air traffic management, in its annual report in 2012 estimated about 110 safety occurrences per million flight hours in 2011 within the European Airspace [4] with an increase of 12% with respect to the previous year. Without any doubts, a better understanding of the limits and criticality of the current ATM is needed in order to design new airspaces capable of sustaining the increase in traffic load.

Air Transportation is a paramount example of an increasingly interconnected techno-social system [5] that requires a strong interoperability of the technological infrastructure with a human and social component. The direct evaluation of the impact of a traffic load increase is of course not feasible for such a system and the only way to explore different scenarios and characterize different development schemes is to resort to theoretical modeling and data-driven simulations. This is nowadays possible thanks to the availability of huge amount of data on which suitably devised computational and modeling schemes can be based. Concepts borrowed from Complex Systems science have been successfully applied in many areas far from traditional physics. Many biological and techno-social systems have been successfully studied and modeled within this framework. Folding proteins [6], social dynamics [7], jamming in vehicular [8, 9] and pedestrian traffic [10, 11] are just some of the many examples of phenomena that have been studied. One of the most recent and successful application of the data-driven modeling approach is the study of epidemic spreading [12–15]. In this process air transportation plays an important role, allowing the infection to spread across distant locations [16]. In the field of transportation systems, many efforts have been devoted to investigate the emergence of finite capacity limits. The Nagel-Schreckenberg model [9] is the most popular model describing vehicular traffic flows that presents a phase transition from a state in which all cars move at high velocity, to a jammed state in which no car can move. Similar models have been developed also for the air traffic, studying phase transitions [17], percolation effects [18, 19], delay propagation [20, 21] and network resilience under random failures [22]. Despite the increasing interest in this subject, no models aiming at describing the ATM system at the level of the trajectories of the single aircraft and their geographical structure have been developed so far, except for a limited part of the airspace [23].

In this paper we present a model of air traffic system at the level of the aircraft trajectories. In our model, the air traffic is regulated by artificial controllers mimicking the real ones of the Air Traffic Control (ATC) system. Their action consists in a dynamical rewiring of the air traffic network over which aircraft are flying. In this sense, the system can be seen as a diffusion dynamics over a dynamical network, which can be locally reshaped when particular traffic conditions occur. Such kind of dynamical networks have been already approached in the literature and appear in very different contexts, from the study of the resilience of the Internet structure [24, 25], to the functional aspects of brain [26]. A general modeling scheme based on suitable communication protocols has been proposed recently to achieve self-healing network structures, i.e., networks that are capable of rerouting the information flow to overcome random or intentional failures [27]. The action of air controllers is similar in behavior to such communication protocols, whereas controllers can rewire the air transportation network in real time.

**Fig 1:**
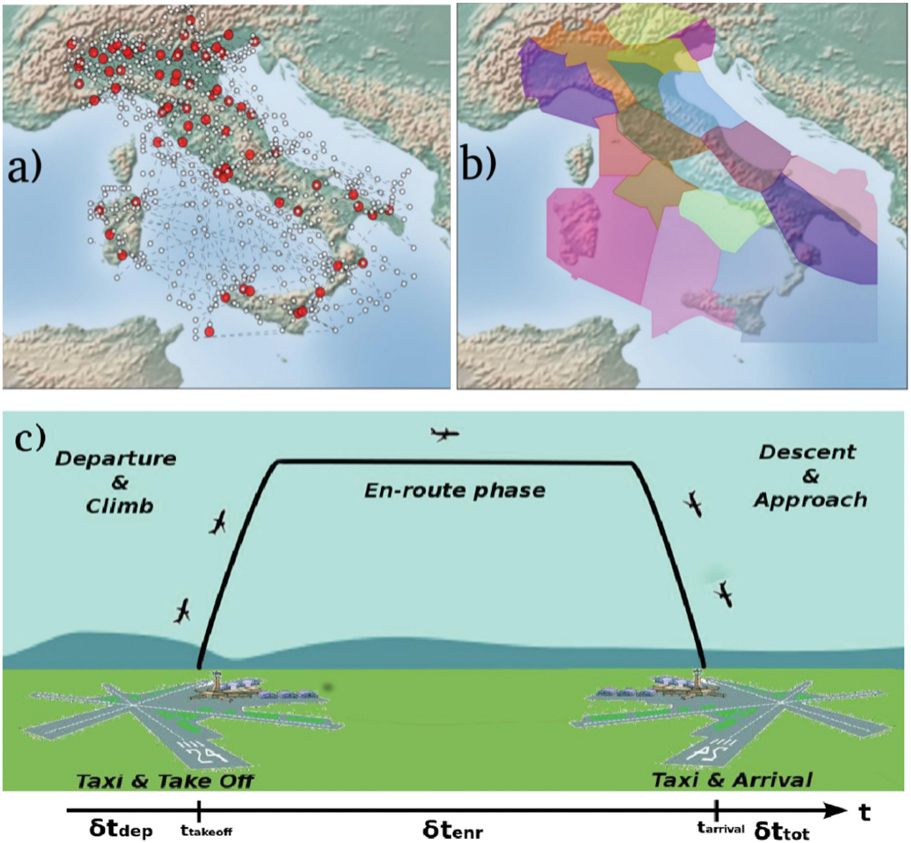
Typical structure of a national airspace and phases of a flight. (a) Navigation point network in the Italian Airspace. White nodes are navigation points, red nodes are Airports. (b) Two-dimensional projection of the sectors in the Italian Airspace during one typical day of operation (sector structures may change depending on traffic load). (c) Phases of a flight: the time line at the bottom indicates the phases of route where the delays *δt*_dep_ at departure and *δt*_enr_ during flight, are generated.

The current ATM system has a very complex and multi-layered structure. Each national airspace is divided in three-dimensional volumes called *sectors* in which controllers take actions to avoid the occurrence of safety events. Inside each sector, aircraft are supposed to fly according to planned trajectories that follow predefined *airways* connecting some geographical references called *navigation points*. As an example, Fig 1 displays the structure of the Italian Airspace both at the level of the navigation points and airways, and at the level of the sectors into which it is divided. The existence of airways is an heritage from the past, since nowadays controllers are helped by advanced technological instruments in their monitoring action. Since the planned trajectories do not take into account the possibility of conflicts with other aircraft and the occurrence of bad weather conditions, the controllers usually have to re-route the aircraft in real time to prevent these risky situations. In fact, they are able to perform the re-routings necessary to provide safety without following the preexisting airways. Their action can be seen as a process of temporary link creation over the network of navigation points, making the structure of such network highly dynamical. Since the proper functioning of the ATC is highly affected by human behavior, it is important to investigate and understand the action performed by controllers in their management activity. For this reason our model simulates, in a stylized way, various strategies of conflict resolution between aircraft used by controllers to prevent safety infringements. We found that as the traffic load increases, the system undergoes a transition from a phase in which all the conflicts between aircraft are resolved to a phase in which many are not resolved anymore. This phenomenon occurs despite the strategy of conflict resolution chosen and indicates that the current ATM system has an intrinsic capacity limit due to the local optimization process performed by controllers. Moreover, we find that the scaling properties of the transition depend on the particular topology used to model the airspace, indicating that the interplay between the topological properties of the network and the action of the controllers can influence the capacity of the system.

Such a situation of air traffic volumes near the transition is hard to achieve in current air spaces. It might be possible that in response to exceptional events (e.g., sudden airport closures or natural disasters) [28] the system could locally reach a state with very high local aircraft densities, but the capacity constraints presently agreed in the ATM system make such *global* unsafe high traffic loads impossible to reach. This prevents us from validating the model by means of actual data close to the transition. Nevertheless, historical flight data can be used to compare the effects of the real ATC on the trajectories of the aircraft to those resulting from simulations performed with our model. In fact, we validate our model by simulating seven full day schedules of flights in the Italian national airspace, by comparing the statistics related to the metrics of the single trajectories and by quantifying the topological changes performed by the ATC over the air transport complex network. The agreement between the model and the measured statistical quantities indicates that the model has a good predictive power and that it can be used as a proxy to study the behavior of the ATC in the usual condition of normal traffic or give sufficiently reliable hints in case of dangerous high traffic load. With these premises in mind, we show that the current airspaces lie below the transition provided that controllers keep using a particularly demanding technique of redirection (the *direct assignments*).

## Materials and Methods

### The data set

The structure of the European Airspace is multi-layered [29], since each national airspace is further subdivided in sectors which in turn group together a certain number of navigation points and airways. In order to study and reproduce this structure, we used information extracted from a database developed within the ELSA project [30]. The database stores data coming from the Demand Data Repository (DDR) [31], which are property of EUROCONTROL, but can be accessed for research purposes on request.

The data includes all the trajectories of civilian and commercial flights that have crossed the European Airspace in the year 2011. There are two kinds of trajectories stored for each flight: the last filed flight plans and the radar updated trajectories.

Last filed flight plans are submitted by the air companies to the Central Flow Management Unit from six months to an hour before the departure of the flight and are the result of the planning process and agreement between these two actors. Instead, the radar updated trajectories are the trajectories that aircraft actually follow after the take off. Both the last filed flight plan and the radar updated trajectory consist of a sequence of geographical coordinates with the time and the height (called *flight level*) at which the aircraft is supposed to cross or really crosses them. Once again, we stress that the last filed flight plans usually do not coincide with the actual trajectory flown. The data delivered by EUROCONTROL are characterized by a variable temporal sample rate of aircraft trajectories, with an average value of about two minutes. Since, as a matter of definition, a conflict occurs when at least two aircraft are closer than 5 Nautical Miles (1 NM = 1.852 km) in the horizontal direction and 1000 feet (1 ft ≈ 300 m) in the vertical direction, the typical time duration of a conflict is about 1 minute considering that the average speed of an aircraft with respect to the ground is about 800 km/h. Thus, the temporal resolution of the data set is not sufficient to spot the possible losses of separation between couples of aircraft, which can be expected to be many in the planned data and very few in the radar updated trajectories. In this paper we will consider all the scheduled flights in Europe from the 8th of June to the 21st of June 2011. Another needed source of information is about the structure of the airspace (i.e., the structure of the sectors inside national airspaces) in each considered day. Such structure may vary according to the traffic demand, since almost every original sector can be split in two or more distinct ones in order to keep the workload assigned to controllers below a certain limit. By using additional data stored into the database, we can in principle extract the shape and boundaries of all the national airspaces and their sectors for every hour of the day, whereas for simplicity we assume that the structure is fixed at the beginning of the day and that it cannot be dynamically reshaped during the operations.

## Results

### Delays

**Fig 2:**
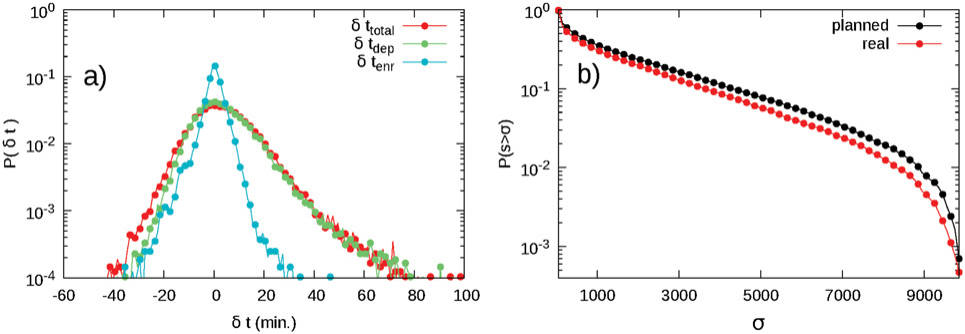
Global effects of the Air Traffic Control (ATC) activity. (a) Histograms of the total, departure and en-route delays for all the aircraft that have crossed the Italian Airspace in the period covered by our data set. (b) Inverse cumulative distribution of the strength of the nodes of the planned and real European navigation point network built by aggregating all the trajectories in the data set.

Let us start our analysis by focusing on the issue of delays. Flight delays are one the primary concerns of the various actors involved in ATM. On one side, delays can damage companies by increasing their costs and can make them miss arrival or departure slots at the airports, on the other, delays can create congestions and unbalances of the traffic load, which controllers have to cope with, and increase the emissions of CO_2_ due to attempts to recover from them. With the data set at our disposal, we can measure the delay *δt*_tot_ of each flight as the difference between the arrival time in the flight plan and in the radar updated trajectory, i.e. the real experienced delay. This delay takes into account all possible sources of delay gathered during all flight phases that we summarize in Fig 1c. We can also measure the departure delay *δt*_dep_ as the difference between the planned departure times and the actual one. We define the difference *δt*_enr_ = *δt*_tot_ − *δt*_dep_ as the *en-route* delay, i.e. the delay generated between the take off and the arrival phase of the aircraft. The histogram of *δt*_enr_ displayed in Fig 2a shows the action of the controllers on the flights. In case of no safety infringements or other external events, this histogram would be highly peaked around the null value. On the contrary, we find that it is still peaked around that value, but it also shows two non negligible tails for negative and positive values of delay with an unbalance toward the negative ones. These tails are the result of the action of controllers who can speed up and delay an aircraft in order to avoid safety events or adverse weather conditions. For example, a large positive en-route delay could be generated after a redirection of an aircraft far from its planned route due to a large weather front that prevents it from following its original path, while negative delays can be generated by controllers trying to shorten the aircraft trajectories in order to decrease the amount of traffic inside the sector they have to manage. The histograms of *δt*_tot_ and *δt*_dep_ look quite similar to one another, indicating that the delay acquired at departure time is the main contribution to the total delay. Interestingly, *δt*_enr_ and *δt*_dep_ are uncorrelated (see Figure B in S1 File), indicating that controllers either are not aware or do not take into account the delay at departure during their management activity and consequently would not try to reduce it.

### Network Analysis

The topological structure of the trajectories in a National Airspace can be recovered by our data set by building a Navigation Point Network (NPN). The topology of such network has been already studied in the case of the Chinese Airspace [32] and recently in the European Airspace [33], using a similar dataset provided by EUROCONTROL [3]. This network is built by using the fixed geographical references as nodes and by linking them if at least an aircraft flew from one to another in the considered period of time. Since we have two sorts of trajectories, it is possible to build two different networks: a *planned* NPN, built with the last filed flight plans, and a *real* NPN built with the actual radar updated trajectories. We point out here that, during the planning session, the actual airways are mostly taken into account so that the study of the last filed flight plans gives hints on the real airway structure, while the real navigation point network dynamically created by controllers during their management action may differ substantially from the airway structure.

We consider weighted networks with two possible natural ways to weight the links, i.e., by using either the geographical distance between nodes or the number of aircraft that flew from a node to another in the considered period. In the latter case the strength of a node (the sum of the weights of the links connected to it) coincides with its traffic load. Thus, the difference between the strength of a node in the planned and real case shows how the traffic load is redistributed over the network by controllers. Fig 2b shows the inverse cumulative distributions of the strength of the nodes for the planned and real NPNs for the whole European Airspace. These distributions have similar exponential-like profiles, though in the real case there are fewer nodes with high load. Practically, the action of controllers results in a redistribution of the traffic load from highly trafficked nodes to lower trafficked ones, making the network more homogeneous in the real case than in the planned one. The distributions of strength are qualitatively similar and the values of strength of the single nodes in the two networks are well correlated (see Figure D in S1 File). This indicates, as expected, that the ATC does not produce global rearrangements of traffic load in the airspace, but just small variations in response to local disturbances such as adverse weather conditions or aircraft separation losses.

The results of the analysis of the delays and the navigation point networks are particularly robust in time and space, meaning that considering a smaller portion of the airspace and a shorter period of time would not change them significantly. Further details can be found in Section A of the Supporting Information S1 File.

### Modeling the ATC

In our model, each aircraft moves on a NPN according to a flight plan made up of a sequence of navigation points to be crossed. These flight plans can be either selected from the historical data extracted from our data set or artificially defined according to some predefined criteria (e.g., the simplest definition of a flight plan is the shortest path connecting the arrival and the destination of the aircraft). The process of redirection made by the controllers is modeled as a local search algorithm applied to the nodes of the NPN. Every redirection performed by the ATC can create a new temporary link connecting two previously unconnected nodes that will be traveled by the redirected aircraft.

The current safety standards state that two aircraft must never come closer than 5 NM in the horizontal direction and 1000 ft in the vertical direction. Whenever these two conditions are violated, the aircraft are considered *under-separated* and a conflict occurs. We would like to model the ATC so to focus only on the en-route phase of the flight, disregarding the taxi, climb and descent phases. Moreover, without being too far from reality, we assume that all aircraft fly at the same constant speed of 800 km/h, so that by neglecting the relatively small vertical component of the flights, we can turn the spatial separation into a temporal one.

**Fig 3:**
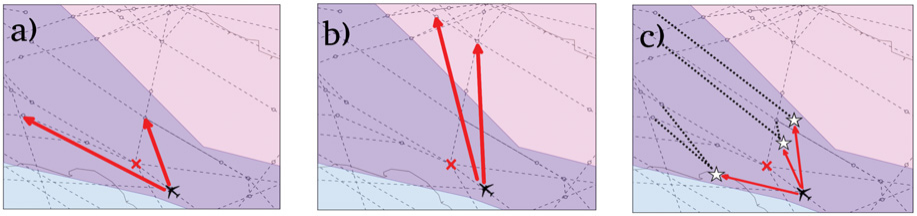
Conflict avoidance strategies. (a) Redirection of aircraft towards nodes inside the current sector, depicted in pink color (IN Strategy); (b) Redirection towards nodes inside nearby sectors (OUT Strategy); (c) Possible redirections towards nodes in the sector that do not belong to the navigation point network (Vectoring). The node highlighted with a red cross is the conflicted node towards which the aircraft is supposed to fly according to its flight plan.

We introduce three strategies of conflict resolution, inspired by the real actions that controllers perform to manage air traffic, i.e., the IN, OUT and *vectoring* strategies depicted in Fig 3. We combine these strategies into three different protocols in order to test their effectiveness in case of high traffic. We call the protocols as IN-OUT, OUT-IN and vectoring-OUT according to the composing strategies and their order of application. In the simulation of the real Italian airspace we used the IN-OUT protocol (see below for more details), which in fact, is the most used by controllers since it requires the least effort, and disregarded the other two. Although the vectoring strategy allows more freedom since controllers redirect aircraft towards any geographical point of the sector, it is the least used because it forces controllers to follow the aircraft singularly in a more detailed way. We considered these strategies as defined over a two-dimensional space, in which the vertical component of the flights is neglected, though vertical deviations can be easily accounted for (see Supporting Information S1 File). In case all strategies of conflict resolution at disposal fail to solve a conflict, the conflict simply occurs without affecting the trajectory of the aircraft. All the strategies and the conditions for loss of separation are discussed in detail in Section B of the Supporting Information S1 File. Note that even though all these results are related to the Italian Airspace, similar simulations can be easily performed in every European Airspace. See [34] for simulation in the Estonian and Greek Airspace, which correspond to the case in which the airspace is small and the boundaries are relevant and the case in which the airspace is at the boundary of the whole European Airspace.

### Data-driven Modeling

By using a data-driven approach we set up a simulation in the Italian Airspace in which flight schedules are real schedules extracted from the data set. Besides the already mentioned IN, OUT and vectoring strategies used by controllers to solve conflict situations, we introduce two more ingredients mimicking the real behavior of controllers, i.e., the *direct assignments* and *sector capacities*. A direct assignment is a maneuver a controller may perform to speed up the traffic and reduce his workload. It is performed by sending an aircraft to a nearby sector, i.e. it relies on an OUT procedure, and requires the coordination of the controllers in the two sectors involved. Although very similar, it differs from an OUT strategy, which is only used to solve conflicts. In our model we simulate the direct assignments by considering a sector dependent probability *p*_direct_ deduced from our data set, so that each time an aircraft crosses a navigation point in its flight plan, it can be directed towards a nearby sector with such probability. This kind of redirection occurs only if it shortens the trajectory of the aircraft allowing it to arrive at destination earlier. Further, in the real-world ATC, each sector has an assigned *capacity*, i.e., an assigned maximum number of flying aircraft per hour that can fly through it. To prevent the possibility of getting very high traffic loads inside a sector, these capacities must not be exceeded during both the planning phase and the management activity of the controllers. In the simulation, sector capacities are taken always into account so that no redirection that would exceed the capacity limits is accepted.

In summary, the simulation of the Italian airspace runs as follows: when an aircraft leaves a node and heads towards the next one the controller chooses among the following strategies in order of importance: (i) with a sector dependent probability *p*_direct_ he assigns a *direct*, but only in case that it shortens the flight and does not cause a conflict; (ii) if the direct is not assigned the controller checks whether in the next node a conflict would arise; if not, the aircraft proceeds to the planned node otherwise (iii) the controller chooses first the IN strategy and if it does not succeed, the OUT strategy (i.e., the IN-OUT protocol). More information on the direct assignment and on sector capacity constraints can be found in Section C of the Supporting Information S1 File. If all these strategies fail, the aircraft goes on straight to the next node, a conflict arises and is recorded.

**Fig 4:**
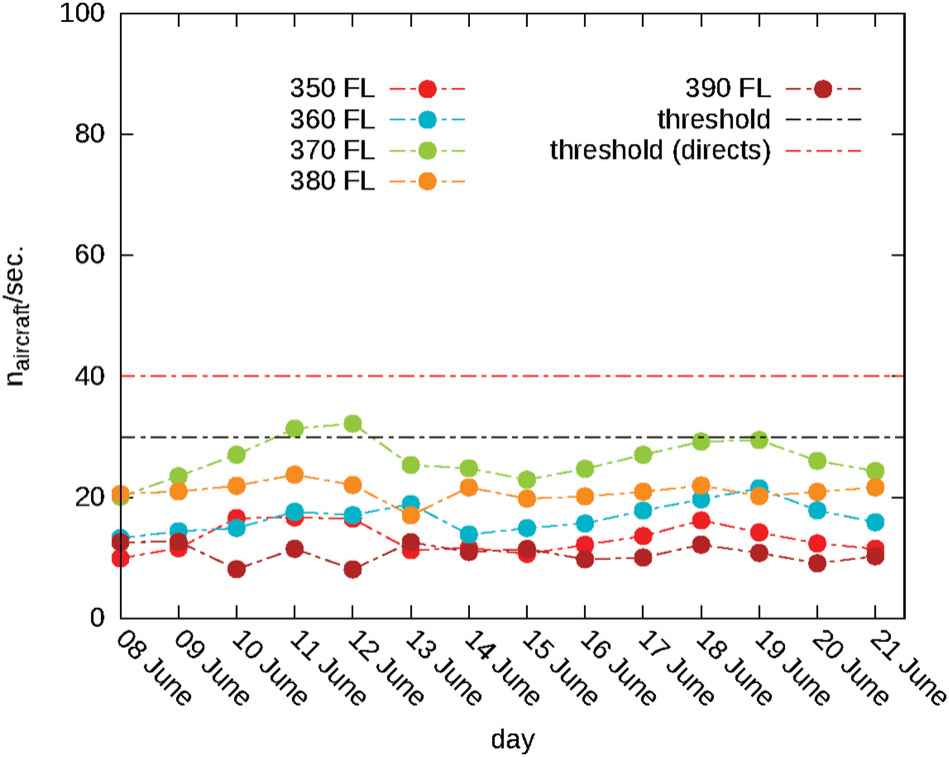
Comparison of actual traffic density with theoretical conflict thresholds. Average number of flying aircraft per second for various days of the dataset in the Italian Airspace. The different curves correspond to different flight levels and their maxima are attained during weekends. The black horizontal dotted line is the estimated threshold value above which the conflict phase emerge with the IN-OUT protocol integrated with flight level changes. The red horizontal dotted line is the threshold with the same IN-OUT protocol with FL changes enriched with the direct assignment procedure.

Finally, since the vertical component of the flights cannot be disregarded in a realistic case, we assigned to each aircraft a desired *flight level* (also inferred from the data set) and allowed small vertical deviations from it. Details on the setup of the simulations used as validation can be found in Section C of the Supporting Information S1 File. The first check performed is that no conflicts are generated in any of the validating simulations done. This is a fundamental condition to assure that the model is reproducing correctly the real situation, where the very few conflicts recorded do not stem from the ATC management. In Fig 4, we show the value of traffic load, above which the system is not able to solve conflicts, with the same protocol used for the validation (dotted red line) and compared it to the actual traffic load inferred from data. We found that the daily traffic load lies below this threshold provided that the direct assignment procedure is also used.

**Fig 5:**
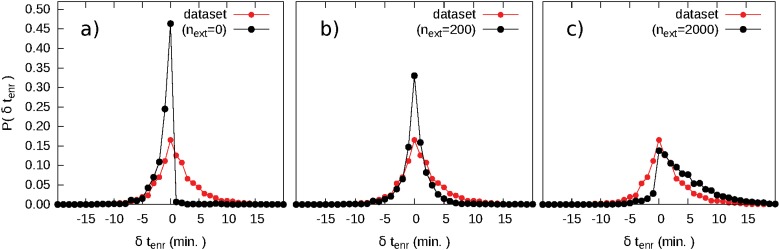
Comparison between the distributions of the en-route delays from data set and simulations. (a) Simulation without external disturbances. (b) Simulation with *n*_ext_ = 200 external disturbances. (c) Simulation with *n*_ext_ = 2000 external disturbances.

As a coarse grained measure of agreement between the simulations and the data set, we compare the distributions of degree, strength and betweenness centrality of the nodes generated by the simulation with the corresponding quantities of the planned and real NPN. In this way, i.e. by aggregating the temporary links created by the ATC in a final static network, we are able to compare the topology and the traffic deployment before and after the action of the ATC. We find a good agreement between the distributions obtained by the simulation and those of the data set. If we take the values of degree, strength and betweenness of the planned network as a reference, we find also a good correlation between the real network and the simulated one, indicating that the model is performing rather well (see Table C in S1 File). Further, we compared the statistics of the single trajectories by measuring their en-route delays, their variation in length and in number of crossed navigation points. While for the last two we find a good agreement between data and simulations, the en-route delay distributions are quite dissimilar (Fig 5). In fact, since redirections are the result of an optimization process, they are likely to produce negative delays instead of positive ones. To reproduce correctly the behavior of the en-route delay distribution, a random external effect affecting the action of the controllers has to be introduced. The ATM system is far from being isolated, since adverse occurrences like bad weather conditions affect the system on a daily basis, and it is already proven that introducing external disturbances can improve the predictive power of an ATM model [35]. Therefore, we introduce in the model some external disturbances as delay generating perturbed areas. The number of such external disturbances, *n*_ext_, is the only free parameter used in our simulation.

From our data we have no clues on the typical number of disturbances during the day therefore we analyzed the response of the model to various values of *n*_ext_. We find that all the measures of topological variation in the NPN and the variations of length and crossed navigation points of the trajectories depend slightly on *n*_ext_, while the shape of the distribution of the delays *δt*_enr_ is very sensitive to it. In Fig 5 it is shown that with a relatively small number of disturbances (≈ 200) the agreement improves, while with a higher number (≈ 2000) the agreement is not recovered.

### High traffic transition in Synthetic and Realistic Airspaces

Since the model is able to reproduce the action of the controllers in a realistic way, our idea is to use it to study the behavior of the ATC in unusual conditions. In particular, we inject an artificial growing traffic load into the airspace and study the effect of the protocols of conflict resolution.

As already mentioned, we defined three protocols (IN-OUT, OUT-IN, vectoring-OUT) composed by a main strategy and a backup strategy. Whenever an aircraft has to be rerouted to solve a conflict the main strategy is used and if it fails, i.e., if no available nodes are found, the backup is used. This assures that the main strategy is mostly used, thus allowing to study its effects individually. Since we aim at increasing the traffic far beyond the actual limits, no capacity limits are assigned to the sectors and no direct assignment is used in these simulations. Moreover, we do not use a 24 hour schedule as before, rather shorter schedules of a fixed number of *n*_aircraft_ aircraft departing in a time frame of 2 hours. Since both the departure time and the flight plan of each aircraft are randomly assigned, we calculated the quantities of interest as an average over 10,000 realizations.

**Fig 6:**
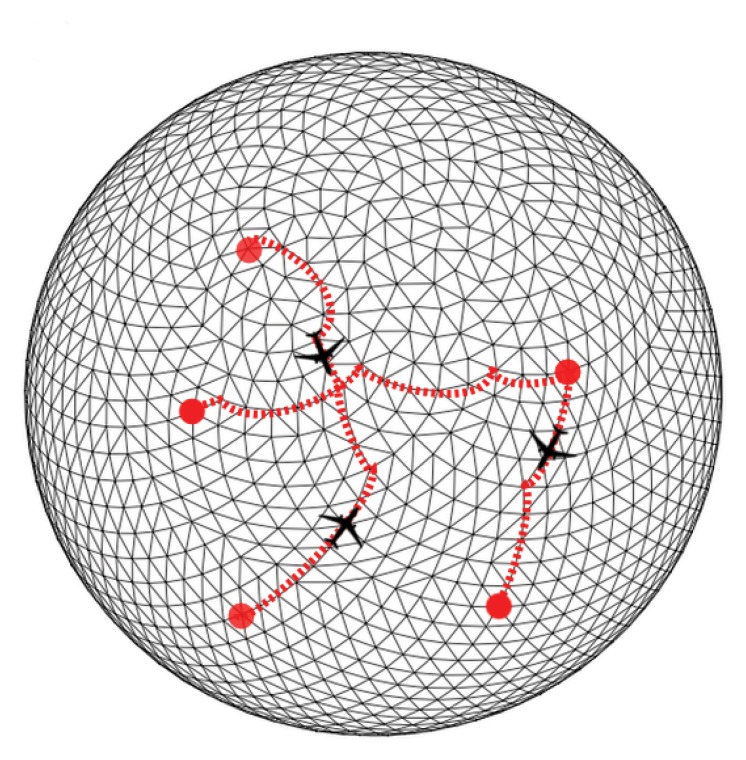
Synthetic boundary-free airspace. Representation of a synthetic airspace, built by using a Fibonacci Grid on a sphere.

We first perform the simulations of traffic growth on a synthetic airspace with periodic boundary conditions. This airspace is embedded on a sphere of fixed radius *R* and its navigation points are built by means of a Fibonacci Grid [36], based on the Fibonacci recursion. The NPN is then built by triangulating the points of the grid, so that in the end we obtain a lattice with coordination number six (Fig 6). Some of the nodes are randomly chosen as airports, which are the starting and ending points of aircraft trajectories, and the surface of the sphere is divided into sectors. We keep the proportion between the surface of the airspace, its navigation points, its number of airports and its number of sectors as close as possible to real airspaces. Such artificial airspace minimizes the finite-size effects due to the absence of a border that could reduce the rerouting possibilities for aircraft flying close to it.

**Fig 7:**
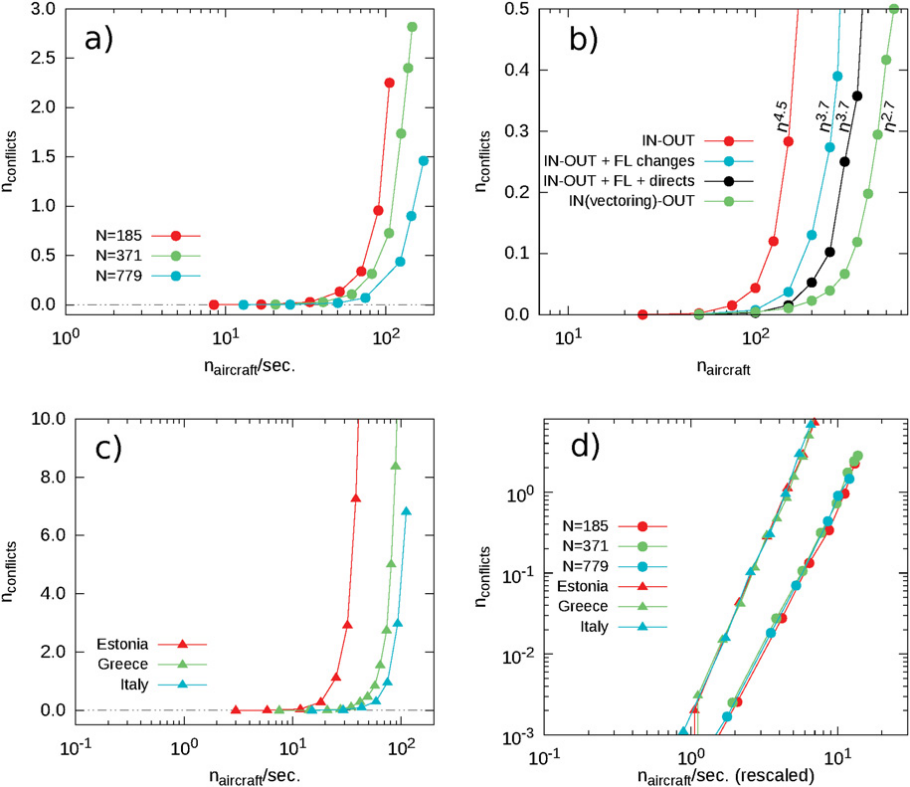
Transition in synthetic and national airspaces. (a) Transition for the IN-OUT protocol in the synthetic airspaces with *N* = 185, *N* = 371 and *N* = 779. (b) Transition for the IN-OUT, Vectoring, IN-OUT with vertical deviations and IN-OUT with vertical deviations and direct assignment in the Estonian airspace. (c) Transition for the IN-OUT protocol in the Estonian, Greek and Italian National Airspaces. (d) Scaling property of the transition for the IN-OUT protocol in the synthetic and real airspaces; in the case of the synthetic airspace the horizontal axis has been rescaled by *N* ^0.40^, while in the real airspace by *N* ^0.43^.

For these airspaces we find a transition from a phase in which all the conflicts are solved to a phase in which many are not. The control parameter of this transition is the average number of flying aircraft per unit time, while the average number of conflicts *n*_*c*_ per realization can represent the order parameter (Fig 7a). In fact, the value of *n*_*c*_ is 0 in the conflict-free phase and starts growing as a power-law with exponent approximately 3.6 immediately above the transition. We find that the transition threshold scales approximately as *N* ^0.40^, where *N* is the number of navigation points in the airspace, airports included.

We find similar results in case of realistic national airspaces, built with our data set (a description of the effects of the boundaries and the way we deal with them can be found in Section B of the Supporting Information S1 File). In particular, we find a similar transition as the traffic load increases with the shape of the transition not changing from the previous case (Fig 7c). In fact, the average number of conflicts still grows as a power-law above the transition and the exponent of this growth is approximately 4.5 right after the transition point. With respect to the previous case, the transition threshold scales slightly differently, approximately as *N* ^0.43^. This indicates that, while the topology of the airspace and the way the traffic is deployed on it does not affect the qualitative behavior of the transition, they affect the way in which the transition scales with the size of the system. The scaling behavior of the transition threshold does not depend on the protocols used by controllers and is only related to the topology of the system.

On the contrary, different protocols do affect the power-law exponent of the number of conflicts above the transition (Fig 7b). For the protocols with only the IN and OUT strategy the exponent of the curve is close to 4.5. This exponent lowers to 3.7 by introducing flight level changes while it drops to 2.7 by implementing the vectoring strategy. However, the transition point, i.e., the traffic load at which the first conflicts are observed does not seem to vary. The decrease of the exponent of the power-law of the number of conflicts above the transition is a natural effect due to the increased possibility of resolving the conflicts by enhancing the strategy repertoire. In particular, the vectoring strategy allows to redirect flights towards any point of the map and enlarges considerably the search space of conflict resolution. Fig 7b also shows the transition curve for the protocol used in Section 1 for the validation of the model.

## Discussion

In this paper we analyzed with the tools of statistical physics, the activity of air traffic controllers, who dynamically address the trajectories of aircraft in real time. In addition, we propose a data-driven model that mimics the action of the air traffic controllers by introducing several strategies of conflict resolution in the form of local search processes over the nodes of a navigation point network. These processes modify the network structure by creating temporary links over which aircraft can travel to avoid risky situations, so that the network is dynamically modified to solve conflicts generated by high local traffic patterns. The model has been developed and tested within the European Airspace, but it can easily extended to national airspaces belonging to other continents and could help to identify functional differences between them. We validate the model by simulating full daily schedules of flights in the Italian Airspace based on the actual planned trajectories, which become real trajectories after controllers action. All the distributions and the topological changes occurring in the Italian navigation point network are reproduced by the model, with the only exception of the distribution of the en-route delays. To increase the agreement in this case, we introduce disturbances in the model in the form of perturbed delay-generating areas that the aircraft can avoid or fly through. The model presented is very general. In this paper we tested it only on the Italian airspace, although we expect the same qualitative results on any national European airspace and on any part of the US airspace.

We also investigated our model predictions when the traffic load injected in the system varies. This is highly relevant in view of the foreseen growth of Air Traffic in the next few years. The most important outcome of these studies is the identification of a transition in the system from a conflict-free phase where all conflicts are successfully resolved to a phase where conflicts cannot be resolved anymore. It is interesting to observe that, based on the flight density at present days, one gets that the current Air Transport system lies well below the transition, though only if controllers employ the special redirection procedure, dubbed direct assignments, consisting in rerouting an aircraft to a nearby sector through a coordination of the controllers in the two sectors involved. The existence of the transition has been initially predicted in a simplified and synthetic airspace with periodic boundary conditions, properly arranged in order to eliminate boundary effects, and then extended to realistic airspaces. The shape of the transition is strategy-dependent but not airspace-dependent, meaning that the same strategy in different airspaces generates the same curve for the transition. This behavior indicates that, despite the management strategy chosen, the system will eventually overcome its capacity limits as the traffic increases. With sufficiently high traffic loads the local optimization process performed by the controllers will start to fail, generating losses of aircraft separation due to high local densities and to the resulting lack of rerouting options. All the defined management strategies are able to reproduce some of the effects of the ATC activity, but thanks to the modular structure of the model, possible refinements could be introduced to further improve realism. A look-ahead time for conflict resolution, sector dependent management strategies and human errors are just some examples of the many possible ingredients that could be implemented and studied. Moreover, the new framework introduced is suited to study and understand the weakness of the current ATM system by simulating and studying its reaction to severe adverse occurrences.

In summary our modeling scheme, built and tested using historical data of the current ATM system, represents a very flexible and modular tool for studies about the performances and the capacity of ATM systems under future scenarios, like the *SESAR* scenario introduced by EUROCONTROL [37]. In this scenario aircraft will fly in a less structured airspace with better planned trajectories, more aware of the system’s capacity and to the requests of the aircraft owners. In all these studies our model could give helpful insights on which modifications of the current system really yield an improvement of safety and performances of flights and which may lead to more instability.

## Supporting Information

S1 File (PDF)
