# Supplementary material for: Congestion Transition in Air Traffic Networks
Source: PLoS One. 2015 May 20;10(5):e0125546. doi: 10.1371/journal.pone.0125546 (PMC4439035; doi:10.1371/journal.pone.0125546)
Supplement: S1 File — (PDF) [file pone.0125546.s001.pdf]

Supporting Information for Congestion transition in Air Traffic Networks

*B. Monechi[1], V. D. P. Servedio[2,1], V. Loreto[1,3,4]
[1]Sapienza University of Rome, Physics Dept., Piazzale Aldo Moro 2, 00185 Roma, Italy
[2]Institute for Complex Systems (ISC-CNR), Via dei Taurini 19, 00185 Roma, Italy
[3]Institute for Scientific Interchange (ISI), Via Alassio 11/C, 10126 Torino, Italy
[4]SONY-CSL, 5, Rue Amyot, 75005, Paris, France

Dataset and Data Analysis

.1 Description and Analysis of the Dataset

The dataset we used stems from the Demand Data Repository (DDR) [1] and has been provided by EUROCONTROL. The information stored by DDR includes many aspects of each single flight, such as the arrival and destination airport, the departure time, the requested flight level, and also technical information like the type of aircraft and the number of people in the crew. It is also possible to identify whether an aircraft belongs to civil aviation, to general aviation or to military aviation. This information has been used to filter out all the military flights since they do usually follow different rules than those of civil aviation. We also filtered out all the trajectories starting and arriving at the same airport and with a requested flight level lower than 2000 ft as they surely do not belong to civil aviation. At the end of this filtering process, we come up with a total number of 345,490 flights in 14 days in the whole European airspace. The number of aircraft per day is quite constant and close to the average value of 24,678 flights per day.

The most relevant information stored in the dataset is that of the planned and real trajectories for each flight. Both the planned and real trajectories are stored as a sequence of segments connecting two geographical points that could be both navigation point or simple temporary points used by the flight during its travel. For each side of a segment the corresponding time and altitude are provided so that it is possible to reconstruct the 3-dimensional trajectory in the airspace. The temporary points are uncommon in the flight plans trajectories but their number is large in the real trajectories, reflecting the fact that aircraft do not stick to the planned airways in their flight.

We also had access to “Nevac” files regarding the structure of the airspace in the corresponding days of the DDR data. In these files the structure of the airways and of the sectors inside national airspaces can be found. Since the structure of the sector is not static but the sector configuration can vary according to the traffic demand, the opening scheme of sectors in each day is present. Note that in our simulations we completely disregard this fact and used the information in the Nevac data to build a simplified and static structure of sectors.

The delay of a single flight can be easily measured by the difference between the arrival time in the real trajectory and the arrival time in the planned one. Similarly it is possible to measure the departure delay as the difference between the departure times of these two trajectories. Since the planned trajectories are last filed flight plans, i.e., they are submitted right before the take-off, it is possible that some of them might have been corrected to take into account some departure delays that consequently cannot be seen from the data. The en-route delay is the difference between the total and departure delay. Note that in

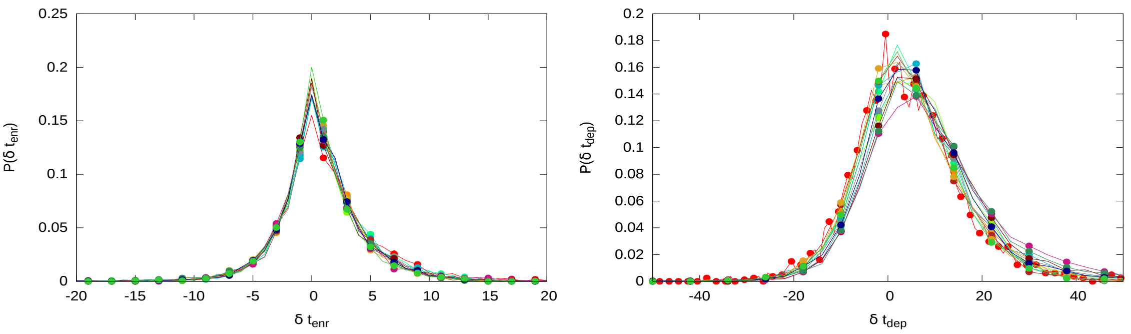

Figure A: Distributions of the delays in every day of our dataset. (Left panel) En-route delay distribution; (Right panel) departure delay distribution. Different colors correspond to different days.

many cases some parts of the real and planned trajectories do not coincide so that it is not possible to define easily a point-wise en-route delay but just a global one at the end of the flight.
The distribution of en-route and departure delays are quite robust in time and space, meaning that in different nations and different days their qualitative features are almost the same. Fig. A shows the distributions of these delays for a week of flights in the Italian Airspace. From one day to another small differences can be observed just in the departure delays distribution, but the en-route one is almost stable. Moreover, these two quantities are uncorrelated in every day of the dataset (the correlation coefficient is always smaller than 0.05), indicating that the delay acquired before the take-off is not managed by the Air Traffic Control (Fig. B). This information may be surprising, but usually controllers completely disregard the delay of a flight when they have to decide to shorten its trajectory or not. All the redirections are applied just for safety reasons or to reduce their workload.

B.2 Analysis of the Navigation Point Network

By using both planned and real trajectories it is possible to built the navigation point networks. These networks are built by considering a certain set of trajectories (e.g., only the trajectories that have crossed a chosen airspace in a chosen time window) and by using the navigation points in such trajectories as nodes of the network as explained in Sec. 2 of the main text.
The planned and real networks have many similar qualitative features. These two networks have similar topological features like an exponential-like degree distribution (Fig. C), probably resulting from the spatial embedding of the network [2, 3], an exponential-like distribution of the strength of the nodes (Fig. 2 of the main text) and of betweenness centrality (Fig. C. In the calculation of the betweenness centrality the links have been weighted by using the geographical distance between the nodes). From these distribution emerges that the real navigation point network is more homogeneous than the planned one, i.e., the action of the controllers reduces the number of nodes with high degree, strength and betweenness, creating more nodes with low values of these metrics. Another similar feature is the shape of the distribution of the weights of the links (Fig. E) that is a power-law with an exponent close to -1.3 and an exponential cut-off. Despite this variations, the values of strength for the corresponding nodes in the planned and real network are well correlated, indicating that the action of the ATC does not produce large changes in how the traffic is deployed over the network (Fig. D).
The two networks have about the same number of nodes, 13,528 nodes in the planned and 14,951 nodes in the real network including the airports as nodes. However, the real network as a number of links that is more than double with respect to the planned case, 44,052 links in the planned and 116,879 in the

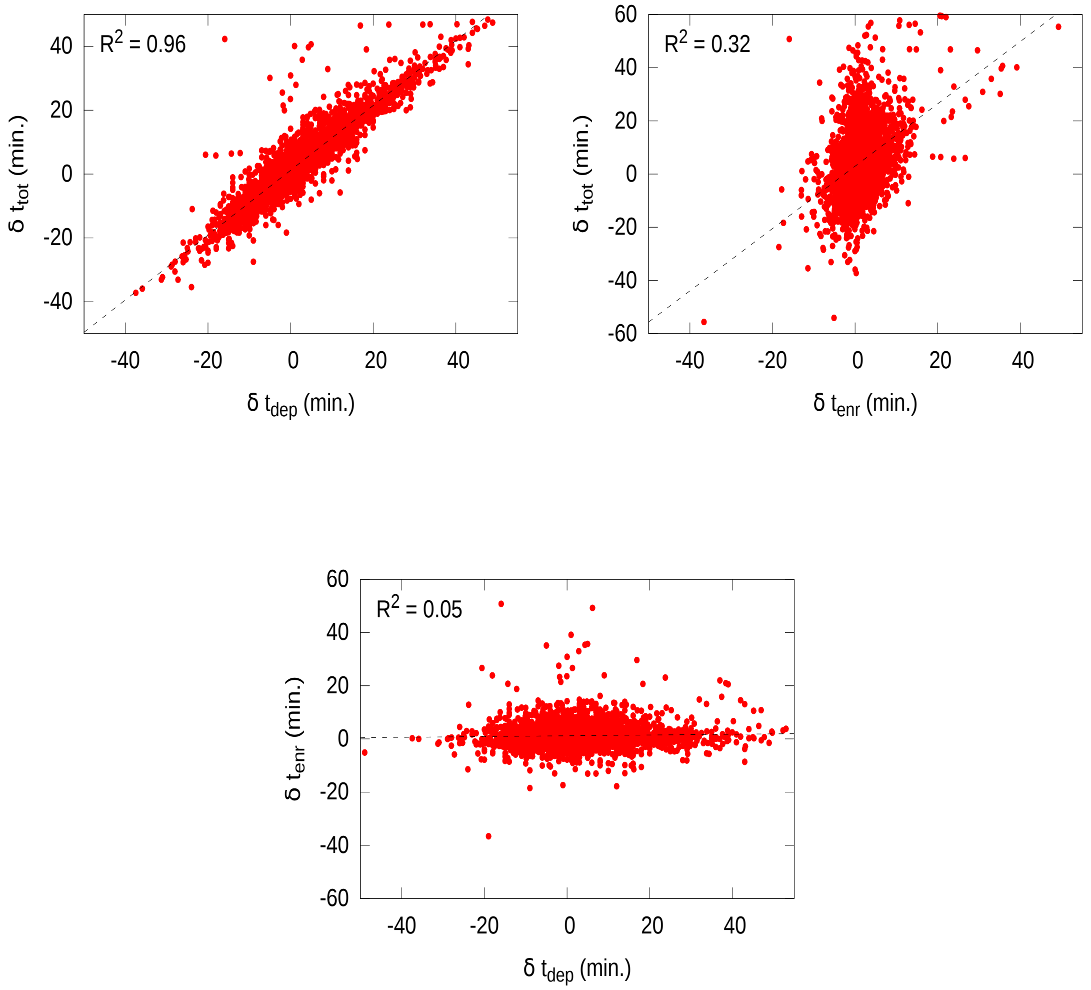

Figure B: Delay correlations. Correlations between the departure delay and the total delay (top left), between the en-route delay and the total delay (top right) and between the departure delay and the en-route delay (bottom). The points correspond to all the flights in the Italian Airspace on the 9th of June 2011.

real case. This is a consequence of the action of the controllers who by redirecting the aircraft create new connections in the network. Moreover, as can be seen in Fig. E, the new links are usually longer than those present in the planned case, since longer connection shorten the trajectories at best. The fact that we have many links longer than the typical linear dimension of a sector (100 NM) indicates that some times aircraft are directed from their current sector to another one without passing through any navigation point. This is a common action performed by the controllers called *direct* that is used to lower

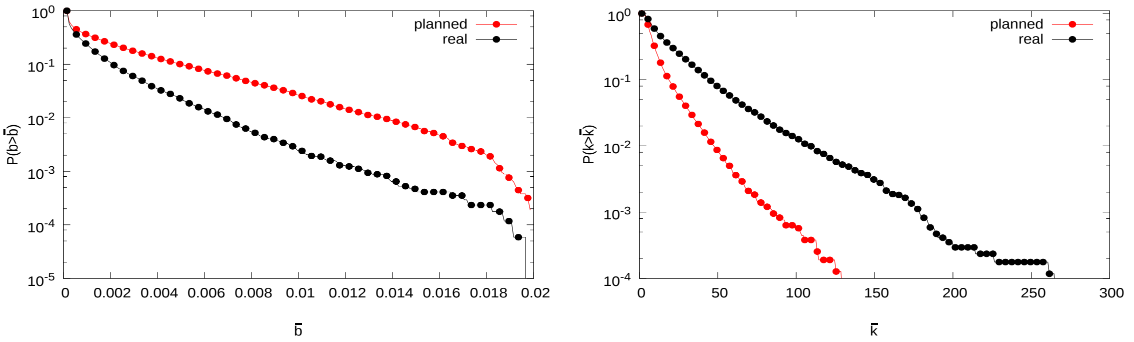

Figure C: Degree and betweenness centrality distributions of airspace networks. Inverse cumulative distributions of degrees (left) and betweenness centralities of the nodes (right). Red curves correspond to the navigation point network built with planned trajectories, green curves to the one built with real trajectories.

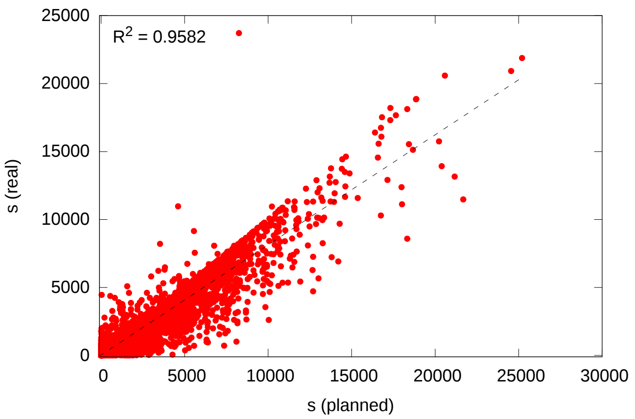

Figure D: Coorelation of node strength. Correlations between the strength of the nodes in the planned European Navpoint Network and the corresponding values of strength in the real European Navpoint Network.

the traffic load inside the sector by sending some aircraft away and thus easing their monitoring activity. Another interesting feature of the real navigation point network is the negative correlation between the length of the links and their traffic load. Fig. E shows a histogram of the scatter-plot between these two quantities. As the distance increases, the binned traffic load starts to decrease, meaning that longer links are harder to be traveled because they are more likely to intersect many trajectories and thus are prone to generating conflicts.

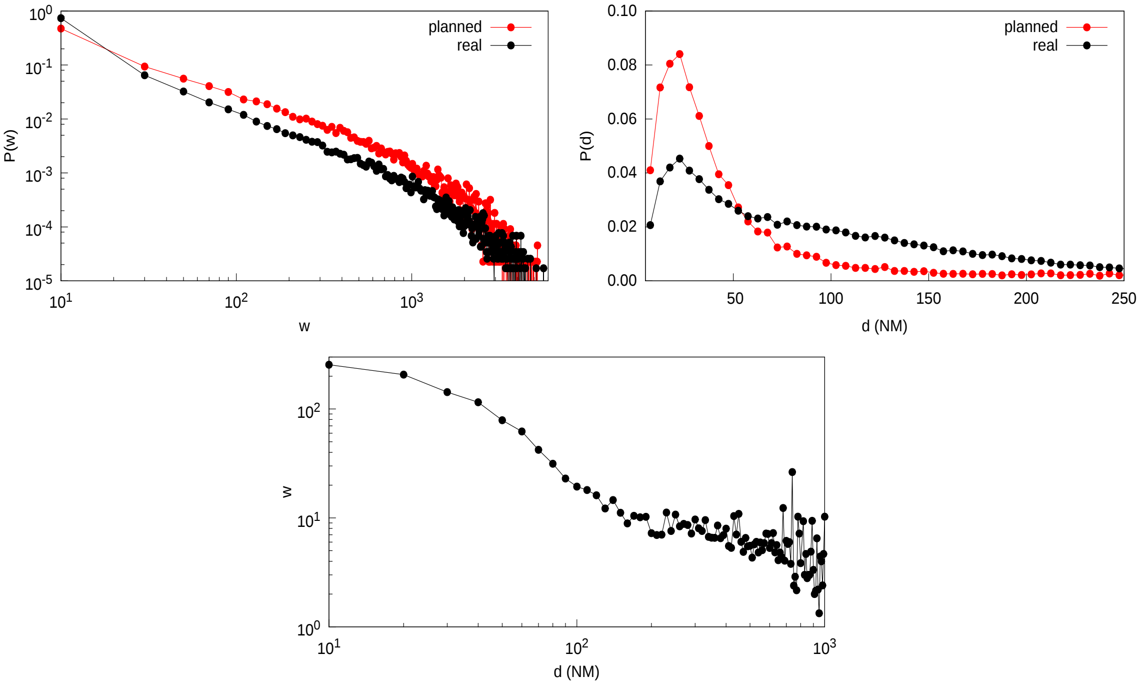

Figure E: Links related quantities. (Upper Left) Cumulative distributions of the link weights (traffic load) for the planned and real European Navigation Point Networks. (Upper Right) Normalized histogram of link lengths for the same two networks. (Bottom) Binning of the scatter-plot between the length and the weights of the links for the real European Navigation Point Network.

E The Model

E.1 Conflicts and Avoidance Strategies

From the analysis of the delays and the navigation point networks two facts are evident: controllers are not aware of the delay of an aircraft and when an aircraft is re-routed the aim is to shorten its trajectory as much as possible. We model the action of the controllers as a local search over a (planned) navigation point network. In our model aircraft fly according to a planned trajectory with a departure time assigned randomly from a uniform distribution in the time frame $[0, 2]$ hours. The planned trajectories consist of a series of navigation point connecting the origin and the destination nodes of the flight, which can be either airports or boundary points of a national airspace. We disregard the take-off and landing part of the flight, so we assume that all the aircraft fly at the same average speed of 800 km/h. These are good approximations for the en-route phase of the flight since variations in speed are rare and not efficient in terms of fuel consumption and horizontal deviations are considered more frequent than the vertical ones. At every time step of simulation (1 s) for every aircraft that has arrived at one of the navigation points in its flight plan, controllers check the next one for conflicts. If a possible conflict is spotted, the controllers apply avoidance strategies trying to redirect the aircraft towards a point of the airspace where possibly no conflict are generated. When all the strategies fail, the aircraft is not redirected and the conflict occurs. The current safety standards for separation between aircraft state that two aircraft must not approach one another less than 5 NM on the horizontal direction and $\approx$ 300 m on the vertical one. If these two conditions are violated, the aircraft are "under-separated" and a conflict occurs. If we do not consider the

vertical dimension, only the horizontal separation must be applied. Moreover, the assumption of constant equal speed of all aircraft allows us to translate the spatial separation into a temporal one. Considering two aircraft i and j, each passing over the same node at the times t_i and t_j respectively (Fig. F top left panel), a conflict occurs whenever

$$
\begin{aligned}
&|t_i - t_j| < \delta\hat{t}(\alpha), \\
&\delta\hat{t}(\alpha) = \max\left(\frac{\delta t}{\sqrt{1+\cos\alpha}}, \delta t\right).
\end{aligned} \tag{1}
$$

In this equation $\delta t = 1\,\mathrm{min.}$ and the factor $(1-\cos\alpha)^{-1}$ is a correction to the separation threshold due to the fact that the conflict can occur after that one of the two aircraft have crossed the node. In fact, supposing that the aircraft i is the first one to cross the node, α is the angle between its exit segment and the segment that the aircraft j is still flying. Note that the threshold becomes infinite if this exit angle is 0, i.e., when the aircraft are heading one towards the other. This condition is applied when two aircraft are flying towards the same node. However a conflict can also occur if one of the aircraft has already passed through a node (Fig. F top right panel). Assuming that this aircraft is the aircraft j a conflict can occur if the following condition holds:

$$
\begin{aligned}
&|t_i + \bar{t}_j| < \delta\bar{t}(\beta), \\
&\delta\bar{t}(\beta) = \max\left(\frac{\delta t}{\sqrt{1 - cos\beta}}, \delta t\right)
\end{aligned} \tag{2}
$$

where $\bar{t}_j$ is the time at which the aircraft j has crossed the node, β is the angle between the two links that the aircraft are flying. Since conflicts can occur also in the intersection between two links and not only over nodes, conflicts on links are also checked in the same way as for the nodes (Fig. F bottom left panel). Moreover, conflicts can occur on segments that do not cross each other (Fig. F bottom right panel). In those cases all the previous conditions for conflict occurrence still apply by considering the intersection of the extensions of the segment as a virtual node, and by considering as crossing times t_i and t_j the times at which the aircraft would have been on the intersection moving on a straight line without changing segment.

Since controllers can redirect aircraft both inside their current sector and towards a nearby one, we defined two main strategies of conflict resolution:

IN: the aircraft is redirected towards a node inside its current sector. This event represents in fact, the redirection that controllers usually perform inside the sector under their responsibility;

OUT: the aircraft is redirected towards a node in an adjacent sector.

The OUT strategy is similar to the so called *directs*, i.e., the controllers may steer a flight directly to another sector, if this does not cause problem in the traffic management. Contrary to the OUT strategy, directs are not used to solve conflicts but just to reduce the traffic load inside a sector and usually require the coordination of the controllers working on the two sectors involved. Whenever a strategy is applied controllers seek for nodes toward which the aircraft can be re-routed without generating conflicts. Among these nodes it is chosen the one that minimizes a cost function defined as

$$
C_0(n, m, s) = d_{n,m} + d^{sp}_{m,s}, \tag{3}
$$

where n is the current node of the aircraft, m is the node that is being considered for a redirection, s is the destination node of the flight, $d_{n,m}$ is the geodesic distance between n and m and $d^{sp}_{m,s}$ is the shortest-path over the network connecting m and s. Since every aircraft has the same velocity all the distances used in the cost function are converted to the time needed to travel them. Since the controllers are not forced to follow the predefined airways to redirect aircraft, redirections between nodes that are not connected in the original navigation point network are possible.

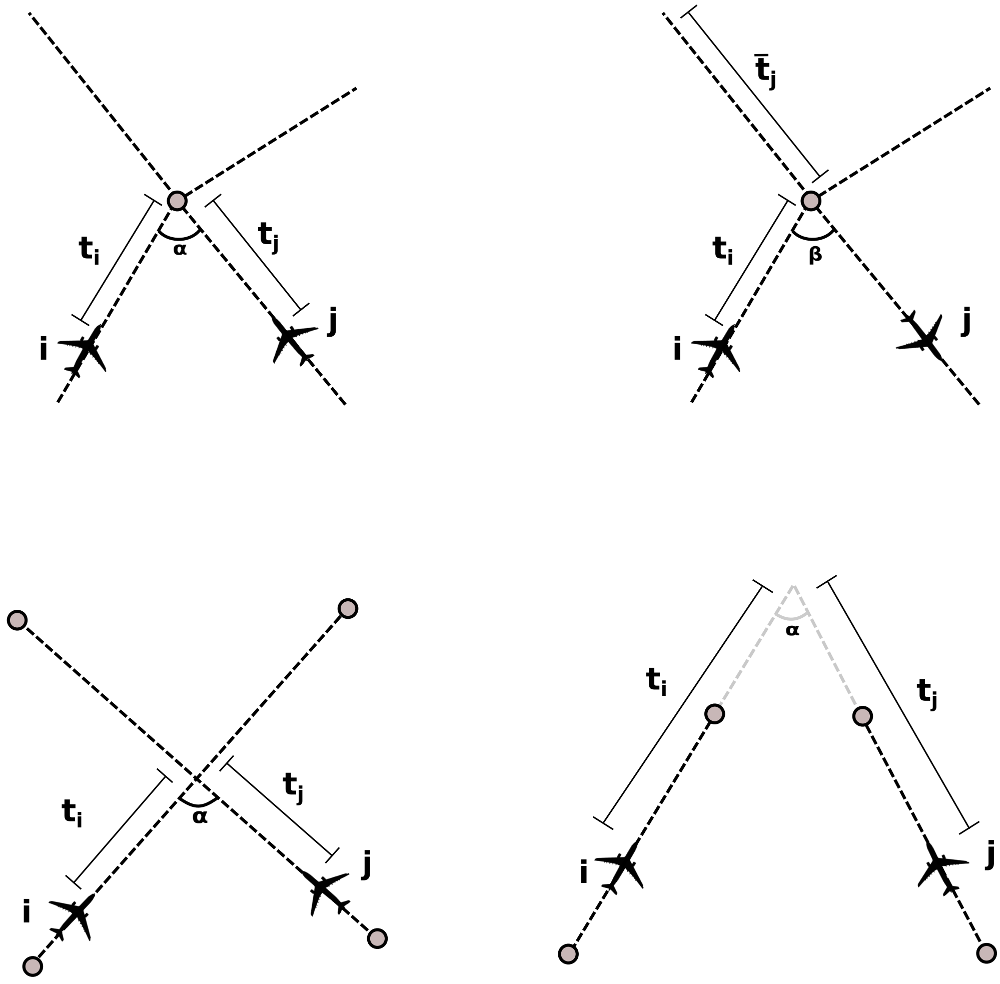

Figure F: Different sorts of conflicts. (Top Left) Representation of a possible conflict occurring to two aircraft flying towards the same node. (Top Right) Possible conflict occurring when an aircraft has already crossed the node. (Bottom Left) Possible conflict occurring on the intersection of two segments. (Bottom Right) Possible conflict occurring on two non-intersecting segments.

F.2 Vectoring and Flight Level

We assumed so far that aircraft fly between nodes always following a straight line. This is a good approximation since, by looking at the new links created in the real navigation point network, more than 80% of them has been traveled in this way. Controllers can actually make aircraft move in a more complicated way, "vectorizing", i.e. addressing, them towards geographical references not present in the network. In order to consider also this possibility, we introduce the *vectoring* strategy:

Vectoring:

some nodes are added to the network inside the current sector of the aircraft in a way that they are not closer to each other and other nodes less than 7 NM. Every temporary node is linked to each other and to every node in the sector. If the conflict is occurring closer than 5 NM from the border of the sectors, nodes are generated also in the next sector of the flight plan of the aircraft. The local search is performed between all the temporary nodes and the other nodes in the sector. At the end of the process all the temporary nodes that are not part of the new trajectory are removed from the network.

The simulations are always performed by using two strategies of conflict resolution, a main one that is used at first and a back-up one that is used whenever the main fails. A pair consisting of a "main and backup" strategy is called a *protocol*. The protocols we use in our simulations are only three, i.e, the IN-OUT, OUT-IN and Vectoring-OUT.
An improvement to both the basic strategies IN and OUT is to add the possibility of flight level (height) changes. During the en-route phase the flight levels available for the aircraft are "quantized" in order to simplify the work for the controllers. The flying level at which an aircraft can fly lies in a range going from 240 FL (where 10 FL $\approx$ 300 m) to 400 FL at discrete steps of 10 FL. In this way two aircraft that are not at the same flight level and that are not descending or ascending are automatically (vertically) separated. We assigned to every aircraft in the system a required flight level, i.e., the flight level that the owner of the aircraft required for his flight. We assume that each aircraft can ascend or descend with the same speed of $10\frac{\mathrm{FL}}{\mathrm{min}}$. Now, each time a horizontal loss of separation is detected also the vertical separation is checked, meaning that the conflict occurs only if aircraft are at the same flight level. The IN and OUT strategies are slightly changed. Whenever controllers have to seek for possible nodes to perform a re-routing, they check all the flight level that are reachable for each node considering the vertical speed of the aircraft and assign them the one that is closest to the required flight level of the aircraft. A strategy fails if there are no available and reachable flight levels in every checked node. Once they found some available node, they chose the one that minimizes a new cost function depending on a parameter $\alpha \in [0, +\infty)$,

$$C_\alpha(n, m, s) = C_0(n, m, s) + \alpha|FL_m - FL_{req}|, \tag{4}$$

where C_0 is defined as in (3) and FL_{req} is the requested flight level of the considered aircraft and FL_m is the closest available flight level on node m to it. The parameter α has been introduced in order to model the fact that in some airspaces controllers may be more reluctant to large flight level changes and prefer horizontal deviations (in that case α is high) and in some other they may care less (α is close to 0). Since the distances used in the cost function C_0 are converted to the time needed to travel them, an $\alpha = 1\ \frac{sec.}{FL}$ with a variation in flight level of 10 FL is equivalent to a penalty of 10 sec. to the total cost function. In other words, since the new term added converts a flight level change in travel time, it can be considered as how much the increase of fuel consumption due to the vertical deviation is taken into account by the controllers.
The IN-OUT protocol with flight level is the conflict resolution strategy used in Section 2 of the main text for the validation of the model, with the addition of capacity constraints and directs (see Section J.1 of this document). Thus, we can use it to test how far is nowadays system from the high conflict phase. Fig. G shows the average number of flying aircraft per second in each day of our dataset. This number has been measured for different requested flight levels. The threshold of the system has been measured as the first value of traffic load in correspondence of which we record at least an unresolved conflict for the IN-OUT protocol with flight level changes and the same protocol with *directs*, introduced in Section 2 of the main text. The two thresholds are the horizontal dotted lines in Fig. G. The traffic loads of the single flight levels are well below the threshold when considering directs. If directs are not allowed, the traffic load is just slightly below the threshold and only in correspondence of a couple of days lies above the threshold in case of $FL = 370$ FL. This result stresses the importance of the direct assignment procedure, since its contribution to keep the system in the conflict-free state is fundamental.

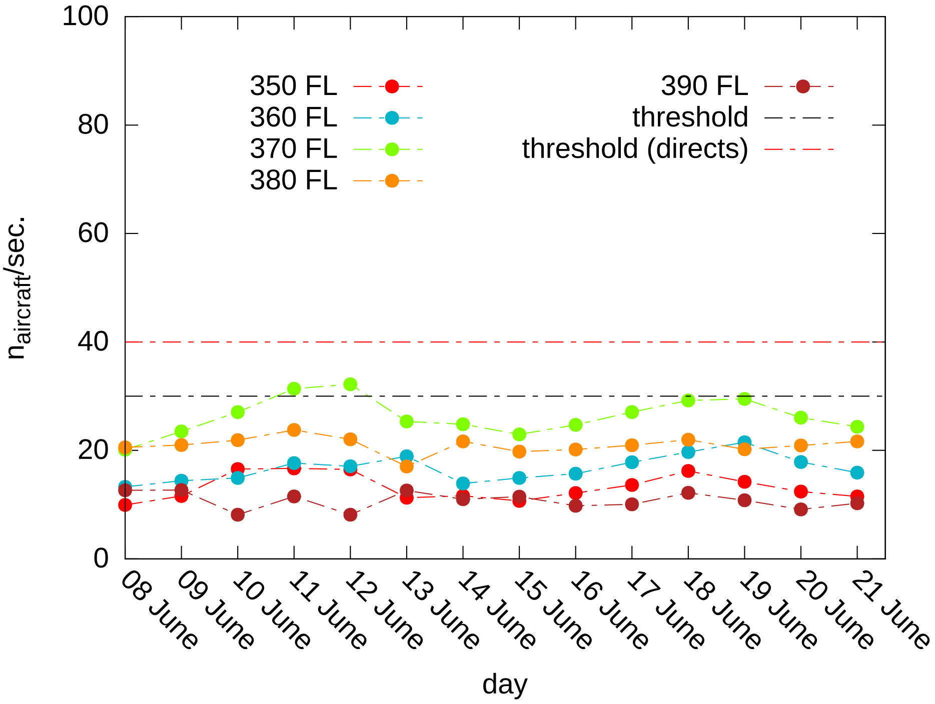

Figure G: Comparison of actual traffic density with theoretical conflict thresholds. Average number of flying aircraft per second for various days of the dataset in the Italian Airspace. Different curves are the values at different flight levels. The black horizontal dotted line is the estimated threshold value that separates the conflict-free and the conflicted phases with the IN-OUT protocol with flight level changes. The red horizontal dotted line is the threshold with the same protocol and direct assignment procedure.

G.3 Synthetic Airspaces

In order to study the model and remove possible finite-size effects inevitably present in realistic networks, we developed a synthetic airspace with no boundary. This airspace is embedded on a sphere of fixed radius R. The navigation points are built by using a Fibonacci Grid [4], based on the Fibonacci recursion. The grid has $N = 2n + 1$ nodes, where $n \in \mathbb{N}$, with latitudes and longitudes defined by the relation

$$\text{lat}_i = \arcsin\left(\frac{2i}{N}\right), \qquad \text{lon}_i = \frac{2\pi i}{\Phi}, \tag{5}$$

where $i = -n \dots n$ are the indexes of the nodes and Φ is defined by $\Phi = 1 + \Phi^{-1}$. The navigation point network is built by triangulating the grid, so that in the end we obtain a lattice of degree 6 (Fig. H). We kept the proportion between the surface of the airspace, its navigation points, its number of airports and its number of sectors making it as similar as possible to real airspaces. Table A reports the number of nodes selected and their corresponding number of airports, number of sectors and surface of the sphere together with the same values for some real planned navigation point networks. With the chosen values, the distribution of the link lengths in the grid is peaked around 28 NM similar to that observed in Fig. E. In order to assign the sectors in the most homogeneous possible way we divide the latitudinal direction in k equal parts, where k is the largest even divider of the chosen number of sectors N_{sectors}, and the latitudinal direction in $m = N_{\text{sectors}}/k$ equal parts. We checked that with the chosen values of N, this

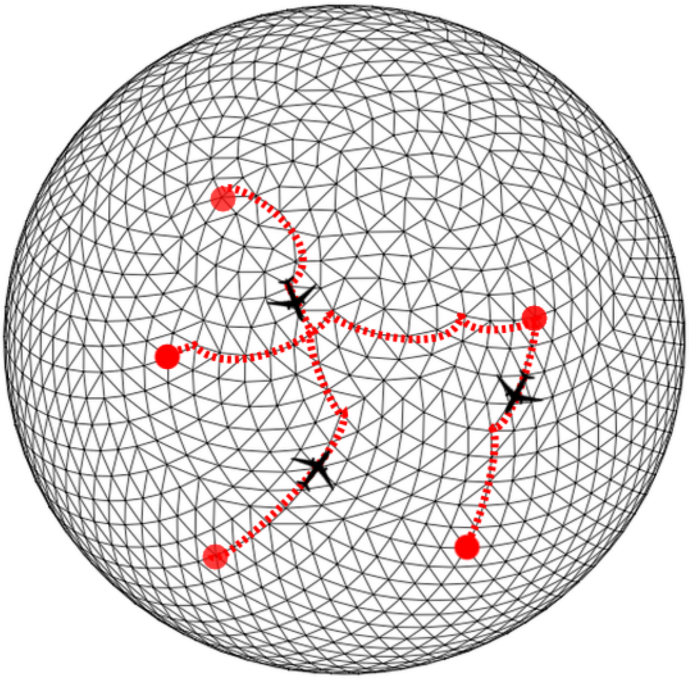

Figure H: Synthetic boundary free airspace. Representation of a synthetic airspace, built by using a Fibonacci Grid on a sphere.

subdivision of the airspace guarantees that each sector contains about 37 nodes. For each realization

Name	N	N_{airports}	N_{sectors}	Surface (NM^2)
Austria	136	5	6	176953
Greece	371	45	9	852729
Italy	725	43	18	1299055
France	1199	63	44	2106885
Synthetic airsp.	185	9	5	425000
Synthetic airsp.	371	18	9	850000
Synthetic airsp.	789	37	20	1784095

Table A: Number of nodes, airports, sectors and surface area of some national arispaces and some synthetic airspaces.

of a chosen value of n_{aircraft}, we randomly chose N_{airports} nodes and use them as airports from which the aircraft departs and at which it arrives. We assume that, from an airport to another, the planned trajectory will always be the shortest-path connecting them. In order to reproduce the fact that some routes may be more exploited than others, we label each airport with different values $k \in \{1, \ldots, N_{\text{airports}}\}$ and use them as "departure fitness". Similarly we assign an "arrival fitness" q randomly reshuffling the departure labels. Considering a flight, the departure airport will be randomly chosen with a probability

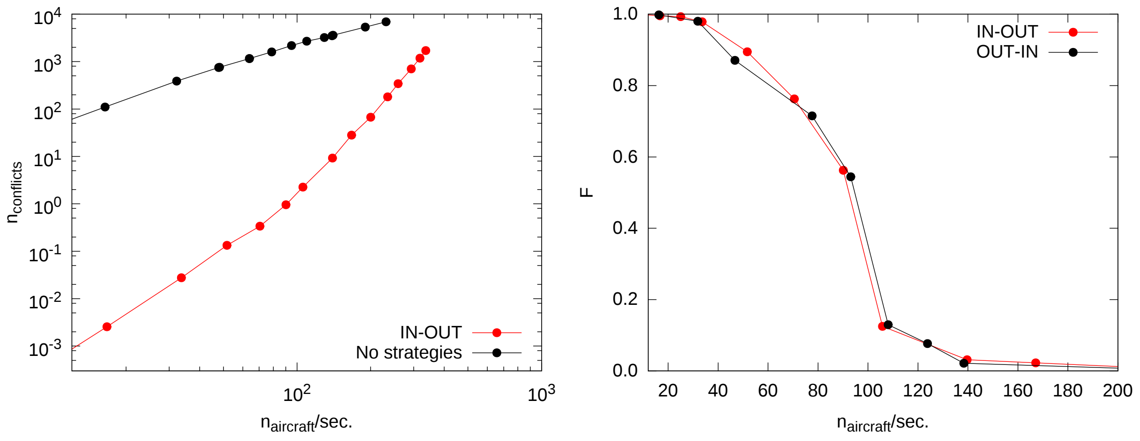

Figure I: Conflicts with and without protocol of conflict resolution. (Left) Number of conflicts as a function of the number of flying aircraft per second for both the IN-OUT protocol and the case in which no redirection is applied. (Right) Fraction of conflict-free realizations as a function of the number of flying aircraft per minute for the IN-OUT and OUT-IN protocol.

$p_{\rm dep} \propto k^{-\tau}$ and the arrival with a probability $p_{\rm arr} \propto q^{-\gamma}$, where τ and γ are always arbitrarily chosen equal to 1 in the following. This is done in order to reproduce the fact that some routes could be preferred with respect to others, while the choice $\tau = \ gamma = 0$ would have meant that the routes are uniformly chosen.
For every protocol defined in Sect. E.1 and Sect. F.2 the model shows a transition from a phase in which all the conflicts are solved to a phase in which many are not. In Fig. 7 of the main text the order parameter used for the transition is the average number of conflicts per realization $n_{\rm conflicts}$. Since we are considering a phase transition generated by the lack of possibilities and not by human error, it has to be expected that at high traffic densities the possibilities of solving conflicts are so few that most of the time the strategies are not used. In that case we expect the system to behave more or less as there were no controllers and conflicts just occur without any attempt of intervention. This is evident by looking at Fig. I in which the comparison between the curves of $n_{\rm conflict}$ for the IN-OUT protocol and for the case in which no strategy is applied. For very high value of $n_{\rm aircraft}$ the curve for the IN-OUT protocol starts to bend and eventually merges with the other one for higher traffic values.

I.4 Realistic Airspaces

Simulation on realistic networks can be built in a simple way by using our dataset. After choosing a national airspace it is possible to use our data to extract the part of the European Navigation Point Network that lies within it. Moreover, it is usually possible to built a simplified sector structure, by projecting the real one onto the earth surface, i.e, the horizontal direction. Note that this procedure cannot be easily performed in every European Airspace. The French Airspace, for example, has a large sector covering large part of the airspace at high altitudes, so a simple projection of its sectors onto the surface of the earth will not lead to a tessellation of the airspace since this big sector will cover many others. However a “hand-made” rearrangement of the sector structure in order to obtain such tessellation is a quite trivial procedure (e.g., in the French case one has just to eliminate the large and high sector). Following what we did for the periodic airspace, we randomly select a certain number of flights and assign them a departure time in a time frame of 2 h. This time we randomly chose the planned trajectories

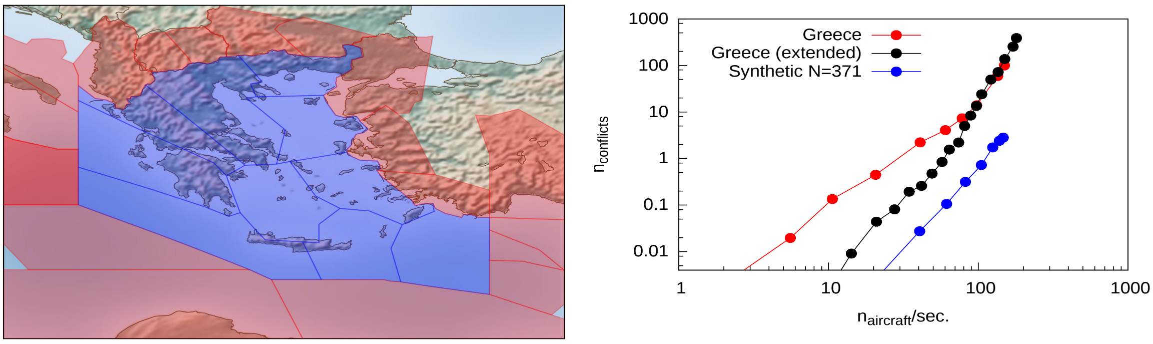

Figure J: Effect of airspace boundary. (Left) Sector structure of the extended Greek Airspace. Blue sectors are part of the airspace, red sectors are used as extension. (Right) Number of conflicts as a function of the number of flying aircraft per minute for the IN-OUT protocol in the Greek, the extended Greek airspaces and a synthetic airspace with the same number of navigation points.

among all the real ones present in our dataset. Note that in this way the most common trajectories will be the most selected, thus assuring that we are reproducing the fact that some routes may be more exploited than others as a result of the demand.
When dealing with realistic airspaces, the effects of the boundaries of the airspace must be taken into account. Suppose that an aircraft has a path that leads to a certain boundary point of the airspace and its getting close to it. When the aircraft is close enough, the possibilities of redirection become very small and it is not possible to redirect it to a node external to the airspace because everything outside it has been cut out and is out of reach. In this case we have an effect of unrealistic enhanced "conflict generation" caused by the presence of the boundaries.
We cured this problem in realistic networks by extending the airspace including not just the sectors inside it, but also those that border on it (Fig. J). We assume that no conflicts can occur in these sectors and that an aircraft that is inside the airspace can be redirected outside only if its next sector in its flight plan is outside the airspace. Fig. J shows the comparison between the transition in the Greek Airspace and in the "extended" Greek Airspace for the IN-OUT protocol. The effect of the boundary has been considerably reduced and both the curves for the extended Greek airspace and the synthetic boundary free airspace show approximately the same slope.

J Validation of the Model

J.1 Setup of the Validation

We validate our model by simulating some schedule of flights in the Italian Airspace, extracted from our dataset. The simulation refers to a lapse of time starting from the 8th up to the 14th of June 2011. Therefore, we simulate both working days and weekends. Each flight plan in the schedule is simulated with its real requested flight level and its real departure delay. We do not have information regarding the further employment of aircraft after arrival (so called "rotation"), so that flights are considered independent from one another and delays cannot propagate from a flight to another one at the airport. In the simulation, the management strategies adopted by the controllers are:

- The IN-OUT protocol is used for conflict resolution, without vectoring;
- Each aircraft starts the simulation already in the "en-route" phase, i.e., flying at its required flight

level;

- Small flight level changes are possible when going from a node to another. Precisely, an aircraft can deviate up to a maximum of 10 FL above and below its required flight level;
- In the cost function of Eq. (4) used for rerouting with vertical deviations, the parameter α is set to 0;
- Each sector has an associated value of "capacity" (C) and "probability of direct assignment" (p_{direct}), both measured from the data, whose values are presented in Table B;
- Each redirection that would violate the capacity of a sector cannot be applied; Each redirection has an assigned new path that the aircraft will follow if the redirection is conflict-free and is the optimal one according to the cost function in (4). This path as a corrensponding series of sector crossing times that can be used to count the number of aircraft per hour that cross each sector. If the redirection make so that this number is higher than the capacity in just one of the sectors along the path, it cannot be applied.
- Each time an aircraft has arrived to a navigation point in its flight plan, the probability of direct assignment is checked. If the direct is assigned, the aircraft is sent to a nearby sector using the OUT strategy; The difference with the OUT strategy itself is that, once a conflict-free redirection with the minimal cost (4) is found, it is applied to the aircraft if and only if it arrival time at destination is smaller than its current one.

The probabilities of assigning a direct are measured as the fraction of aircraft that has gone from a node inside their current sector, towards a node in another sector without passing through any other navigation point and such that the the distance between them is larger than 80 NM at least one time in its current sector (the typical dimension of a sector). We checked that for each considered sector the fraction of directed aircraft was about constant in every hour of the day for each day in our dataset, so we assumed that all the probabilities of direct assignment are constant during the simulation.
Since we do not have any certain information about the real assigned capacities of each sector, we decided to estimate them from our dataset. Considering a sector we measured the number of flying aircraft per hour in each hour of our dataset and use the maximum between these values as its capacity. We can now assume that, as long as capacities are not exceeded, human errors are practically excluded.

Name	p_{direct}	C
LIPPC3	0.07	75
LIPPD5	0.16	66
LIRRTS2	0.24	70
LIPPB5	0.05	144
LIRRMIE	0.13	188
LIRRNW2B	0.05	69
LIRREW2A	0.09	72
LIBBES3	0.09	51
LIRRES2	0.18	54

Name	p_{direct}	C
LIRRNE3	0.09	108
LIRRSU2	0.16	48
LIBBND4	0.18	56
LIBBMD1	0.07	53
LIRRUS1	0.07	81
LIPPE3	0.15	59
LIRROV1	0.16	125
LIPPA6	0.05	115
LIRRMIW	0.10	167

Table B: Probability of direct assigment and capacity of each sector within the Italian Airspace as used in our simulation.

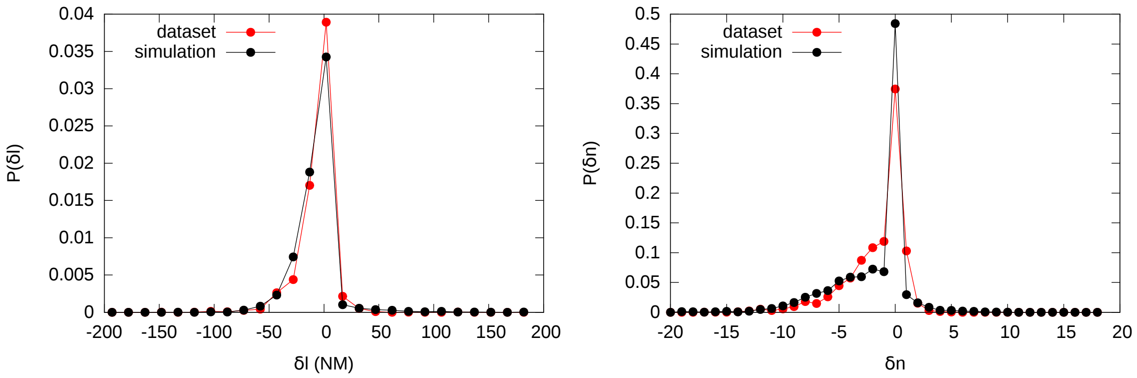

Figure K: Comparison between radar updated and simulation generated trajectories. (Left) Distributions of the variations in length (δl) and (Right) variations in number of crossed navigation points (δn) between the planned trajectories and the radar updated ones and between the planned trajectories and the trajectories generated in our simulations. These figures correspond to the 9th of June 2011.

J.2 External Disturbances

To simulate external disturbances we introduced the first free parameter of the model, i.e., the number of external disturbances $n_{\rm ext}$. Each disturbance is a round area with a randomly assigned radius $r_{\rm dist}$ in a range between 1 NM and 15 NM. Every time an aircraft is flying on a link that crosses one of the perturbed areas it gains a random penalty delay from a uniform distribution in a range between 1 min and 0.14 $r_{\rm dist}$ min/NM, whereas 0.14 min/NM is the inverse of the fixed velocity of the aircraft. Note that if a link crosses more than one disturbance, the delays are cumulated. This in order to model the fact that it is not convenient to fly over highly perturbed areas. Every disturbance is static and affects all the flight levels inside its area. To reproduce the fact that weather changes during the day, every 60 min of simulation the displacements and dimensions of the disturbances are randomly reassigned. The values of $n_{\rm ext}$ used are 0, 200 and 2000 for each day of simulation.

J.3 Results of the Validation

Besides the distribution of the en-route delays, there are other distributions related to the trajectories that can be checked, the variation in length and the variation in number of crossed navigation points. Similarily to the distribution of the en-route delays, the distributions of these quantities are quite robust in each day of simulation. Fig. K shows the comparison between these distributions measured by using the trajectories of our dataset and those stemming from our simulations. We found a good agreement between these distributions indicating that the redirections applied in our simulation are likely to modify the trajectories in a similar way of the action of the controllers. As explained in the Sect. B.2, the action of the controllers generates new links in the network, making the real network denser than the planned one. Table C presents the number of links in the planned and real navigation point networks, built from the 8th to the 14th of June 2011, together with the corresponding number of links in the real navigation point network built by using the output trajectories of our simulations. We found that the number of links are always very similar, with a slight systematic overestimation in case of the simulated network.
Moreover, the distribution of the length of the links of the generated network is in good agreement with that of the real network presented in Fig L, meaning that our model reproduces correctly the length of the new links created by the controllers. In Fig E we presented the anti-correlations between the length and the weight of the links in the real (European) network. This anti-correlations are a common

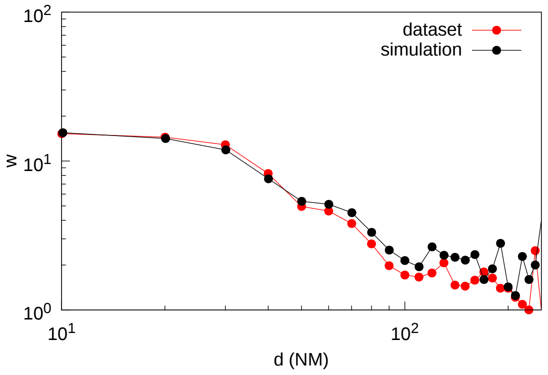

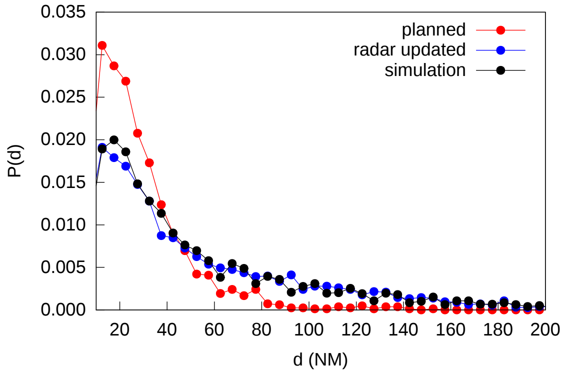

Figure L: Comparison between planned, radar updated and simulated networks. (Left) Binning of the scatter-plot between the length of the links (d) and the number of aircraft that travelled over a link (w) for the Italian Navigation Point Networks built with radar updated trajectories and with trajectories generated by our simulation. (Right) Distribution of the lengths of the links for the Italian Navigation Point Networks built with planned, radar updated trajectories and with trajectories generated by our simulation. These figures correspond to June 9th, 2011.

phenomenon in most airspaces in Europe and the Italian one is not an exception. Again our model is able to reproduce what has been inferred from data (Fig. L). Fig. M shows the distributions of the variations in degree (δd), betweenness centrality (δb) and strength (δs) between the real and the planned navigation point networks. In the same figure the same distributions obtained by building the real navigation point network with the output trajectories of our simulation are also shown. The action of controllers usually yields an increase of the degree of the node, up to an increment of about 20 new connections in some cases. The increment of the number of links, instead, generates new shortest-paths in the network leading to a decrease of the values of centrality of the nodes. Finally, the distribution of the variations in strength is highly peaked around 0 with a slightly longer tail towards negative values, meaning that the action of the controllers naturally tries to decrease the traffic load over the nodes without creating new highly trafficked ones. We checked that the variations of these metrics between the real and the planned navigation point networks were correlated with those between the "generated" and the planned navigation point networks. In all the cases positive correlations have been found, meaning that the model also reproduces the topological variations between real and planned trajectories. Table C presents the correlation coefficients for the variations in betweenness centrality, degree and strength of the nodes for each day of simulation. All the coefficients are quite constant through the days, the correlation coefficient for the variations in betweenness centrality of the nodes is usually the highest (about 0.8), while the other are always larger than 0.5.
All the measures shown in this paragraph correspond to a number of external disturbances $n_{\rm ext} = 100$. However, contrarily to what happens for the en-route delays, these measures do not depend sensibly on the number of external disturbances and the presented results remain valid for a broad set of its values.

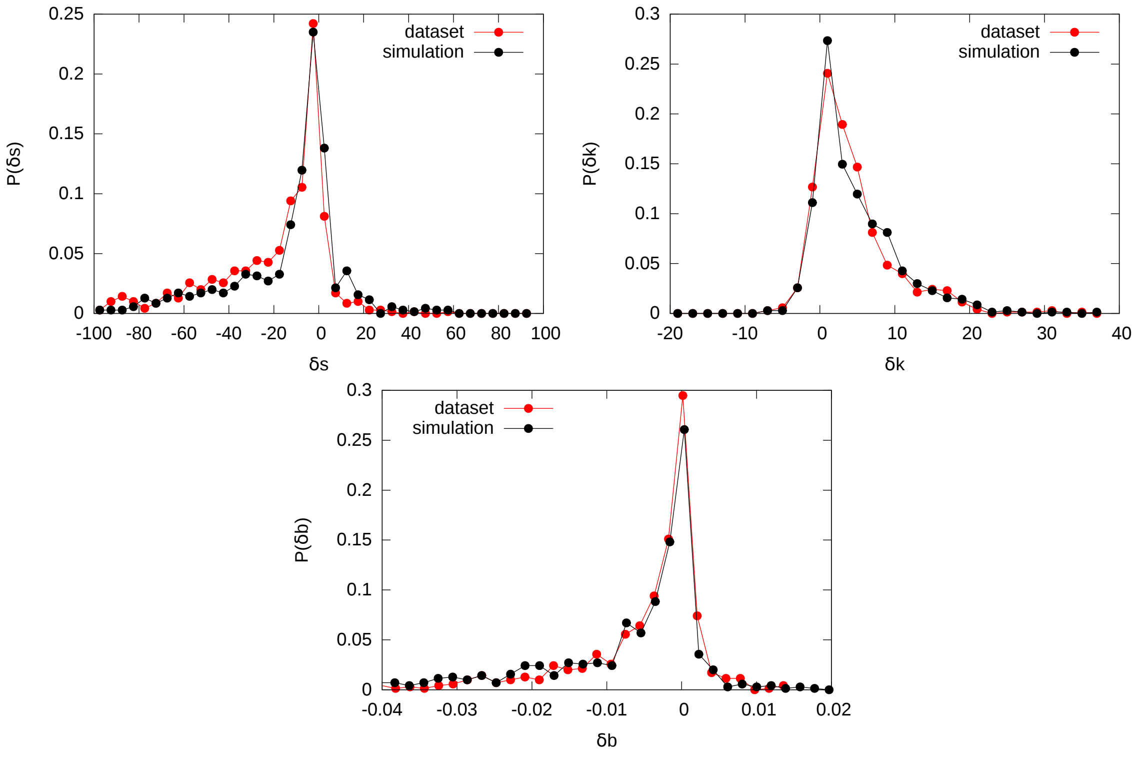

Figure M: Comparison between planned, radar updated and simulated networks. Distribution of the variations in strength (δs), degree (δk) and betweenness centrality (δb) between the Italian Navigation Point Network built with radar updated trajectories and the trajectories generated by our simulation. These figures correspond to the 9th of June 2011.

Days	n_{links} (data)	n_{links} (simulation)	δd corr. coeff.	δs corr. coeff.	δb corr. coeff.
08/06/2011	3318	3151	0.64	0.67	0.86
09/06/2011	3056	3160	0.60	0.81	0.87
10/06/2011	3522	3598	0.66	0.69	0.87
11/06/2011	3323	3419	0.66	0.71	0.86
12/06/2011	3562	3251	0.67	0.64	0.88
13/06/2011	3178	3350	0.64	0.67	0.83
14/06/2011	3163	3230	0.65	0.69	0.84

Table C: Comparison between the real navigation point networks built with the data and with the trajectories generated by simulations, for each day of simulation.

References

[1] EUROCONTROL. Demand data repository reference manual. 1.5.8 edition (Restricted Audience), 2010.

[2] M Barthélemy. Spatial networks. *Physics Reports*, 499(1-3):1–86, 2011.

[3] Alain Barrat, Marc Barthélemy, and Alessandro Vespignani. The effects of spatial constraints on the evolution of weighted complex networks. *Journal of Statistical Mechanics: Theory and Experiment*, 2005(05):P05003, May 2005.

[4] Richard Swinbank and R. James Purser. Fibonacci grids: A novel approach to global modelling. *Quarterly Journal of the Royal Meteorological Society*, 132(619):1769–1793, 2006.
